# Short-term general, gynecologic, orthopedic, and pediatric surgical mission trips in Nicaragua: A cost-effectiveness analysis

**DOI:** 10.7189/jogh.11.04024

**Published:** 2021-05-22

**Authors:** Keyanna P Taylor, Anna Ortiz, Jason Paltzer

**Affiliations:** 1 Department of Public Health, Baylor University, Waco, Texas, USA; 2 Esperança, Phoenix, Arizona, USA

## Abstract

**Background:**

Short-term surgical missions facilitated by non-governmental organizations (NGOs) may be a possible platform for cost-effective international global surgical efforts. The objective of this study is to determine if short-term surgical mission trips provided by the non-governmental organization (NGO) Esperança to Nicaragua from 2016 to 2020 are cost-effective.

**Methods:**

Using a provider perspective, the costs of implementing the surgical trips were collected via Esperança’s previous trip reports. The reports and patient data were analyzed to determine disability-adjusted life years averted from each surgical procedure provided in Nicaragua from 2016-2020. Average cost-effectiveness ratios for each surgical trip specialty were calculated to determine the average cost of averting one disability-adjusted life year.

**Results:**

Esperança’s surgical missions’ program in Nicaragua from 2016 to 2020 was found to be cost-effective, with pediatric and gynecology surgical specialties being highly cost-effective and general and orthopedic surgical specialties being moderately cost-effective. These results were echoed in both scenarios of the sensitivity analysis, except for the orthopedic specialty which was found to not be cost-effective when testing an increased discount rate.

**Conclusions:**

The cost-effectiveness of short-term surgical missions provided by NGOs can be cost-effective, but limitations include inconsistent data from a societal perspective and lack of an appropriate counterfactual. Future studies should examine the capacity for NGOs to collect adequate data and conduct rigorous economic evaluations.

Surgical procedures can be crucial for managing numerous health conditions; however, surgery is often neglected in the arena of global public health. The Lancet Commission for Global Surgery reports that there remains a largely unrecognized and urgent need for surgical care within the world’s poorest populations [1]. It is estimated that in 2010 alone, conditions requiring surgical care resulted in 16.9 million lost lives, or 32.9% of all deaths worldwide [2]. Furthermore, lifesaving surgical interventions could prevent 77.2 million disability-adjusted life years (DALYs) each year [3]. This unmet need for surgical procedures is highest among lower-and-middle income countries, specifically the poor, rural, and marginalized [1]. This often means that among the most vulnerable populations, the lack of simple surgical procedures can be fatal. The Lancet Commission for Global Surgery also states that the need for equitable surgical efforts is only set to increase in years to come as low-and-middle income countries (LMICs) undergo an epidemiological transition wherein cancer, cardiovascular diseases, and road traffic injuries are expected to exceed the previous main challenge of communicable diseases [1]. Therefore, access to and delivery of equitable and quality surgical efforts in LMICs must be a global health priority.

Scaling up surgical care in LMICs might also lead to improvements in national incomes and economic development. Conditions requiring surgery contribute greatly to the global economic burden with this impact being greatest among LMICs [4]. From 2015 to 2030, it is estimated that 128 countries may face a potential cumulative gross domestic product (GDP) loss of US$20.7 trillion or 1.25% due to unmet surgical needs [4]. When considering welfare losses, it is estimated that diseases contributed to US$14.5 trillion losses in 2010 alone [4]. Elevating global surgery as a priority can avert lives lost due to preventable surgical diseases as well as alleviate the macroeconomic impacts of conditions requiring surgery to support economic development in LMICs [4].

However, in recent years promising research has shown that surgical interventions are often cost-effective and therefore should be considered on the global public health agenda [5]. For example, a systematic literature review by Grimes and authors analyzed twenty-seven articles conducting cost-effectiveness analyses of surgical interventions within 64 LMICs and found that the majority of surgical interventions were very cost-effective [6]. Interventions including cataract surgery, inguinal hernia repairs, male circumcision, emergency cesarean sections, and cleft lip and palate repair were found to be cost-effective [6]. Therefore, it can be said that global surgery can be a cost-effective priority effort to prevent long-term disability in low resource settings.

Short-term surgical missions are a common type of short-term medical service trip (MST) which are defined as trips in which medical provider volunteers from high-income countries (HICs) travel to LMICs for periods ranging from 1 day to 8 weeks to provide health care [7]. Faith-based and non-faith based non-governmental organizations (NGOs) an facilitate MSTs and their teams of personnel can consist of individuals affiliated by academic departments or institutions, organizations, or social groups [7]. Short-term surgical missions may be a possible platform for cost-effective international global surgical efforts. Therefore, in the present study, an economic evaluation was conducted to answer the question about the cost-effectiveness of an international volunteer surgical program in Nicaragua from 2016 to 2020. This study’s objectives were to 1) assess the costs of surgeries provided by short-term surgical teams; 2) Determine the DALY for surgeries conducted; and 3) Calculate the average cost-effectiveness ratio (CER) for primary groups of surgeries. The purpose was to increase knowledge regarding the cost-effectiveness of short-term surgical missions provided by a non-profit global health organization which may serve as a potential platform to reduce the disease burden of surgical conditions in LMICs.

## METHODS

A cost-effectiveness analysis (CEA) is a type of economic evaluation that examines both the costs and the health outcomes associated with one or more interventions [8]. A CEA is often used to compare the effectiveness of multiple interventions or compare an intervention against a counterfactual (the status quo) [8]. Conducting a CEA can help policy and decision-makers when selecting health interventions.

Esperança is a non-profit global health organization based in the southwest United States which serves low resource communities through disease prevention, education, and treatment efforts. The health intervention in this economic evaluation is Esperança’s international volunteer surgical program in Nicaragua. In Nicaragua, surgical capacity in primary hospitals and overall surgical availability at the departmental, regional, and national levels is promising [9]. However, lack of human resources and necessary equipment and technology are major contributors to limitations in surgical capacity [9,10]. The provision of subspecialty care in Nicaragua is often limited leading such care to be often provided by NGO short-term brigades [9]. Esperança’s volunteer surgical program in Nicaragua supports teams of physicians and health professionals who embark on short-term mission trips to provide free surgical consultations and procedures to community members. The mission trips in Nicaragua focus on multiple surgical specialties including general, pediatric, gynecology, orthopedics, plastics, dental, and ophthalmology. Participants in the intervention are men, women, and children who receive free surgical consultations and treatment from Esperança’s international surgical mission trips.

The analytic perspective for this analysis will be a provider perspective. Resources, such as costs, and health effects will be identified and valued from the perspective of the health care providers. In this analysis, providers included Esperança, the US team of surgical volunteers, and the local hospital team.

In this analysis, the counterfactual will be a control intervention of no surgical trips being implemented and no procedures being provided by Esperança in Nicaragua or the local health care team from 2016-2020. This is a logical counterfactual because although surgical care in Nicaragua is often free or affordable, it remains a resource poor setting lacking surgeons, subspecialty care, and infrastructure making surgical care more difficult to obtain in Nicaragua [9,10]. However, due to data constraints, this control intervention was not able to be quantified for the analysis. Therefore, the results of this analysis will be presented as an average cost-effectiveness ratio of the intervention costs and health effects. The implementation period of this analysis is from 2016 to 2020. Therefore, the health intervention will be evaluated over these five years. Lastly, the horizon of analysis will be the duration of the lifetime of all intervention beneficiaries. Therefore, the horizon of analysis will be the remaining lifetime years of all patients impacted. Future benefits and future costs are discounted using a 3% discount rate, as recommended by World Health Organization and Shrime and authors [11,12]. All costs are also inflated to 2020 US dollars to ensure that costs across the implementation period are comparable. MS Excel software (Microsoft Inc., Seattle, WA, USA) was utilized for all calculations in this analysis.

Trips were included in the analysis if the necessary data was available such as patient-specific information, provider costs, and the in-kind donation value. Trips were included if the surgical team only provided procedures of a single specialty. No previous data was available regarding the cost-effectiveness of the surgical program; therefore, this analysis was conducted to inform future planning for Esperança’s international surgical program. The analysis is a single study using the available surgical data.

The Consolidated Health Economic Evaluation Reporting Standards (CHEERS) guidelines were followed and each item of the CHEERS checklist was included in this analysis [13]. An institutional review board exemption was submitted to Baylor University’s institutional review board which deemed this study as non-human subjects research.

### Outcomes

The World Health Organization (WHO) Guide to Cost-Effectiveness Analysis describes utilizing DALYs as a unit of measure for cost-effectiveness analyses for various health interventions [11]. Furthermore, Esperança conducts surgical trips to Nicaragua to provide treatment of surgical conditions for individuals who would otherwise not receive treatment. The surgical trips provide relief to individuals with surgical conditions who determine that they cannot afford necessary procedures or do not have access to other medical treatment options. Therefore, surgical procedures were intended to decrease or eliminate disability and illness due to various conditions. Because this is the overall goal of the surgical program, DALYs were an effective outcome to measure the years of disability averted from the intervention. DALYs are a calculated measure to assess the overall burden of a disease and [11] is a sum of years of life lost (YLL) due to premature mortality and years lived with disability (YLD) due to the health condition [11]. The basic equation to calculate DALYs can be seen below.

*DALY = YLL+YLD*

*YLL = N × L*

*YLD = I × DW × L*

When calculating YLLs, *N* represents the number of deaths due to a condition and *L* represents standard life expectancy at age of death in years. When calculating YLDs, *I* represents the number of incident cases, *DW* represents the disability weight, and *L* represents the average duration of the case until remission or death in years. Additional social weights are often included in these calculations such as discount rates, and age-weighting.

Disability weights represent the magnitude of health loss associated with a specific health state and are measured on a scale from 0 to 1, with 0 equating to full health and 1 equating to death [14]. Disability weights were determined by searching for the weight for the health condition associated closest to the surgical procedure conducted for each patient. Weights were collected from the 2017 Global Burden of Disease (GBD) study. However, disability weights specific to surgical conditions are often sparse and difficult to determine [12]. Therefore, additional sources, such as previous GBD studies and other published CEA studies, were used to determine disability weights applied in this analysis. If an exact disability weight was not available, a substitution was made for a disability weight with the closest possible health state. All surgical conditions and the associated disability weights used can be found in Appendix S1 of the **Online Supplementary Document**.

If patients were treated for more than one condition, a disability weight was obtained for each condition they were treated for. To account for comorbid or multimorbid disability weights, they must be combined multiplicatively [15]. This combined disability weight was then used to calculate the averted DALYs. Multiplicatively combining disability weights was done by using the equation seen below.

*DW_Total_ = 1 – [(1 – DW_1_) × (1 – DW_2_) × (1 – DW_3_)]*

While age-weighting was previously endorsed by the WHO Guide to Cost-Effectiveness Analysis, since 2010 the GBD studies no longer utilize age-weighting. It is currently recommended to not use age-weighting in the primary cost-effectiveness analysis [12].

Surgeries provided resulted in no deaths and no surgical complications resulting in additional sequela were reported, therefore YLLs were not included in the DALY calculation. DALYs were determined by calculating YLDs each patient and summing them for each specialty included in analysis. The basic equation for calculating YLDs changes when taking into account social weights such as discounting. The final formula used to calculate DALYs averted can be seen below.

*DALYs Averted = [1 × DW × (1 – (1 – e^–rL^)] / r*

For the equation above, *I* is the number of incident cases, *DW* is the specific disease disability weight, *r* is the discounting rate, and *L* is the average duration of disability measured in years. This was calculated for each surgery and summed for each specialty. Therefore, *I* for each surgery was simply 1. The average duration of disability was assumed to be the remainder of life for each patient, so *L* was determined to be each patient’s age-specific life expectancy based on the WHO Life Tables for Nicaragua in 2016 [16]. An example calculation for DALYs averted can be found in Appendix S2 of the **Online Supplementary Document**.

### Costs

Types of estimated intervention costs in this analysis can be seen in Table 1. Intervention costs were collected from reports for each mission trip, these included costs to Esperança, the US surgical team, and the local surgical team. Provider costs were determined prior to this analysis by Esperança and solely considered the costs for providing services and not opportunity costs. For some trips, transportation and meal costs were not available. In these cases, the estimated costs used were Esperança’s budget estimation for the flight and meal costs.

**Table 1:** Types of costs for Esperança’s Volunteer Surgical Program in Nicaragua

Esperança	
Fixed costs – capital	Facility maintenance/cleaning
	Laundry
	Fuel
	Telephone
	Radio announcements
	Utilities
	Warehouse rent for medical supplies
Fixed costs – labor	Mission coordinator
	Anesthesiologists assistant
	Surgical assistant
	OR nurse
	Assistant nurse
	Guardsman
	Driver
	Translator
Variable costs	Medicine
	Laboratory exams
	Materials/supplies
	Medical complications
	Miscellaneous
	Meals/supplies for the local team
	Office supplies
	Local medical team lodging
Providers:	
Fixed costs – capital	N/A
Fixed costs – labor	In-kind donation services of US surgical team
	In-kind donation services of the local surgical team
Variable costs	In-kind donation materials/supplies provided by US team
	In-kind donation materials/supplies provided by the local team
	In-kind donation laboratory exams provided by the local team
	Lodging for US team
	Meals for US team

Surgery sizes, from small to extra-large surgeries, were determined by medial personnel and are associated with varying estimated values of donated services necessary for procedure completion for both teams. Esperança has previously determined the cost of donated services of the US and local surgical teams. For the US team, the estimated costs of donated services for small, medium, large, and extra-large prodecures were US$1150, US$2070, US$3335, and US$4600, respectively. For the local team, the estimated costs of donated services for small, medium, large, and extra-large prodecures were US$575, US$1035, US$1668, and US$2300, respectively. Surgery size for each procedure in this intervention was determined by the medical personnel conducting the procedures. For each trip, the number of surgeries of each size are determined and used to calculate the overall estimated cost to both teams for the donated service of providing surgical treatment.

All intervention costs were discounted to their present value using the predetermined discount rate of 3% and inflated to 2020 US dollars using the US Bureau of Labor and Statistics Consumer Price Index tool [17]. A discount rate of 3% was used and applied to discount the costs of implementation each year to determine the discounted total costs using the equation below. In the equation for discounting total costs below, c_t_ represents the costs in year t and r equals the discount rate [12].


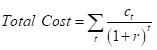


No decision model was utilized for this analysis. This is because all surgical trips were included in the analysis if the necessary procedure and cost data were available. After determining the total estimated costs and total estimated health effects of the intervention, an average CER was calculated. The CER was determined by calculating the average cost per DALY. The WHO defines interventions as highly cost-effective if the CER is less than the country’s GDP per capita, cost-effective if the CER is between one and three times the country’s GDP per capita, and not cost-effective if the CER is more than three times the country’s GDP per capita [11]. This method was used to determine the overall cost-effectiveness of Esperança’s surgical program in Nicaragua from 2016 to 2020. According to the World Bank, Nicaragua’s GDP per capita in 2019 US dollars was US$2107.57 [18]. Similarly to the intervention costs, this value was inflated to 2020 US dollars using the US Bureau of Labor and Statistics Consumer Price Index tool [18]. Once inflated, Nicaragua’s GDP per capita in 2020 US dollars was US$2133.49 and was the value used in determining the cost-effectiveness of the intervention.

### Assumptions

This analysis relied on three main assumptions. First, a 3% discount rate assumed for costs and benefits in the primary analysis. This assumption of a 3% discount rate was tested in the sensitivity analysis. Second, conditions treated by the surgical procedures conducted were estimated by the author. Surgical procedures were researched via the web-based peer-reviewed literature, medical resources, and international health documents and the most common and relevant associated health condition was used to select disability weights. Third, for some surgeries, disability weights for their health condition were easily located. However, overall disability weights were difficult to find, therefore multiple procedure types and their associated conditions were regarded as general or unspecific health states for which disability weights were available.

### Sensitivity analysis

Two one-way sensitivity analyses were run by altering the discounting rate. For the first sensitivity analysis scenario, a discount rate of 0% was conducted to examine the impact on the cost-effectiveness ratio of applying no discounting to costs or effects. In the second sensitivity analysis scenario, a 6% discount rate was used. It has been suggested that the generally applied social discount rate for global health of 3%, is more in line with the economic growth of high-income countries such as the US [19] Therefore, the large difference of economic growth for LMIC implies that a discount rate of at least 5% is more appropriate for CEAs in LMICs, such as Nicaragua [20]. Additionally, WHO CEA guidelines recommend testing a discount rate of 6% in the sensitivity analysis [11].

## RESULTS

In total, 16 surgical trips were included in this analysis. General and gynecology were the most prevalent specialties with 5 trips of each being included in this analysis. Additionally, two pediatric trips and three orthopedic trips were included.

Over the implementation period of the intervention, a total of 563 surgical procedures (258 general, 70 pediatric, 129 gynecological, and 106 orthopedic) were provided. The average trip length was 7.6 days, with a 7.6-day trip average for general surgical trips, an 8-day trip average for pediatric surgical trips, a 6.8-day trip average for gynecology surgical trips, and an 8.67-day trip average for orthopedic surgical trips. The average age of patients treated was 38.04 years old, with the average patient age for general, pediatric, gynecology, and orthopedic trips being 39.97, 5.67, 49.79, and 36.80 years old, respectively. The intervention treated a total of 200 (36.4%) male patients and a total of 363 (66.2%) female patients. Patient and surgical details are summarized in **Table 2**.

**Table 2:** Surgical trip details

Surgical specialty	Average trip length (days)	Average patient age (years)	Number of procedures	Male, N (%)	Female, N (%)
General	7.6	39.97	258	94 (36.4%)	164 (63.6%)
Pediatric	8	5.685	70	43 (61.3%)	27 (38.6%)
Gynecology	6.8	49.79	129	0 (0%)	129 (100%)
Orthopedic	8.67	36.80	106	63 (59.4%)	43 (40.6%
Overall (total)	7.6	38.04	563	200 (36.4%)	363 (66.2%)

The total estimated cost of the intervention was US$3 342 646.90, with specific costs for general, pediatric, gynecology, orthopedic specialties being US$1 367 466.20, US$420 811.72, US$751 376.55, and US$781 233.72, respectively. For general, pediatric, gynecology, and orthopedic surgical specialties the number of DALYs averted was 525.56, 283.15, 431.81, and 125.71, respectively. The estimated costs and DALYs averted for each specialty yielded CER which can be seen in **Table 3**. The CERs reveal that the general and orthopedic surgical specialties of Esperança’s surgical mission program from 2016-2020 were cost-effective. Furthermore, the pediatric and gynecology surgical specialties of Esperança’s surgical mission program from 2016-were found to be highly cost-effective. No known variations exist in the patient populations by each surgical specialty that may explain differences in the cost-effectiveness ratios.

**Table 3:** Cost-effectiveness analysis results

Surgical specialty	Estimated costs*	DALYs averted	Cost-effectiveness ratio (CER)†	Cost-effectiveness results
General	1 367 466.20	525.56	2601.90	Cost-effective ^‡^
Pediatrics	420 811.72	283.15	1486.16	Highly cost-effective ^§^
Gynecology	751 376.55	431.81	1740.07	Highly cost-effective ^§^
Orthopedics	781 233.72	125.71	6214.67	Cost-effective ^‡^

*Monetary values are presented in 2020 US$.

†Cost-effectiveness ratio (CER) is equal to estimated costs in US$ divided by DALYs averted.

‡Cost-effective if the CER > gross domestic product (GDP) per capita (US$2106.567) but ≤ three times the GDP per capita (US$6322.701).

§Highly cost-effective if the CER < GDP per capita (US$2106.567).

The results of the sensitivity analysis can be found in **Table 4** and **Table 5**. In scenario one, all surgical specialties were highly cost-effective except for orthopedics which was generally cost-effective. Similarly, in scenario two all surgical specialties were cost-effective except for orthopedics which was not cost-effective.

**Table 4:** Cost-effectiveness analysis sensitivity analysis Scenario One Results (0% discounting)

Surgical specialty	Estimated costs*	DALYs averted	Cost-effectiveness ratio (CER) ^†^	Cost-effectiveness results
General	1 467 382.09	952.23	1541.00	Highly cost-effective ^§^
Pediatrics	441 196.77	688.53	640.78	Highly cost-effective ^§^
Gynecology	803 799.80	765.46	1050.08	Highly cost-effective ^§^
Orthopedics	845 829.34	235.53	3591.15	Cost-effective ^‡^

*Monetary values are presented in 2020 US$.

†Cost-effectiveness ratio (CER) is equal to estimated costs in US$ divided by DALYs averted.

‡Cost-effective if the CER > gross domestic product (GDP) per capita (US$2106.567) but ≤ three times the GDP per capita (US$6322.701).

§Highly cost-effective if the CER<GDP per capita (US$2106.567).

**Table 5:** Cost-effectiveness analysis sensitivity analysis Scenario Two results (6% discounting)

Surgical specialty	Estimated costs*	DALYs averted	Cost-effectiveness ratio (CER) ^†^	Cost-effectiveness results
General	1 278 438.90	337.15	3791.86	Cost-effective ^‡^
Pediatrics	401 994.96	158.24	2540.35	Cost-effective ^‡^
Gynecology	705 151.48	282.98	2491.88	Cost-effective ^‡^
Orthopedics	723 349.65	80.92	8939.29	Not cost-effective

*Monetary values are presented in 2020 US$.

†Cost-effectiveness ratio (CER) is equal to estimated costs in US$ divided by DALYs averted.

‡Cost-effective if the CER > gross domestic product (GDP) per capita (US$2106.567) but ≤ three times the GDP per capita (6322.701).

§Highly cost-effective if the CER < GDP per capita (2106.567).

## DISCUSSION

Esperança’s surgical missions’ program in Nicaragua from 2016 to 2020 was found to be cost-effective relative to Nicaragua’s GDP in 2020 US dollars. The pediatrics and gynecology surgical specialties were found to be highly cost-effective, with pediatrics having the lowest and gynecology having the second lowest cost per DALY averted. General surgery and orthopedic surgery were both found to be cost-effective with general surgery having the second highest and orthopedic surgery having the highest cost per DALY averted. These results were echoed in both scenarios of the sensitivity analysis, except for the orthopedic specialty in scenario two which was found to not be cost-effective.

The results of this study contribute to the field of literature regarding economic evaluations of international surgical missions provided by non-governmental organizations. First, the results of this study confirm other findings of cost-effectiveness analyses of global surgery, especially of short-term surgical missions. For example, a systematic literature review found that 27 missions in nine LMICs from 2006-2014 with specialties ranging from neurosurgical, orthopedic, and hand surgery were all very cost-effective [21]. Second, this study highlights how a higher discount rate for LMICs may significantly impact cost-effectiveness results. When a higher discount rate was applied, differences in cost-effectiveness across surgical specialties emerged, specifically orthopedic surgery. Orthopedic surgeries were found to be the least cost-effective and were not cost-effective when a higher discount rate was applied. These results highlight specific differences in the cost-effectiveness of various surgical specialties, as seen by orthopedic missions which had much higher intervention costs for the number of DALYs averted. As recommended by Haacker et al. and echoed in these results, the use of a higher discount rate for interventions in LMICs should be considered as this may influence the true results of cost-effectiveness [20].

The results of this study highlight the potential minimal cost-effectiveness when a higher discount rate is applied, which aligns with other studies that have found that short-term surgical missions may not actually be as cost-effective as often reported. For example, Shrime and authors found that cost-effectiveness analysis results indicating that short-term surgical trips are cost-effective, must be accepted with caution [2]. This is mainly because average cost-effectiveness ratios for short-term surgical trips are especially biased towards the provider organization as they do not compare the surgical intervention to another intervention. When short-term surgical missions are compared against alternative surgical platforms, they are often much less cost-effective or not cost-effective [2]. This study suggests that there may be more nuance involved with determining which surgeries are cost-effective depending on the overall county’s capacity to provide health care in determining counterfactuals and control groups.

Evidence from the previously mentioned studies which argue that short-term surgical missions are not cost-effective may be explained by a larger issue when evaluating charitable medical platforms. Lack of rigorous research on short-term surgical missions is a major contributing factor to these findings. The true impact of short-term medical trips is widely unmeasured [7]. Majority of studies examining short-term medical missions provide low-level data when compared to established research evaluation methods [7]. The contributions made by research studies in this area may be limited mainly due to a lack of priority for research among the service provider organizations and the lack of standardized evaluation tools and methods [7]. Research is not commonly a major priority of medical service organizations; therefore; increasing the focus on data collection for medical service organizations may be difficult but critical to realistically evaluate practices and improve health care delivery [7].

The lack of data regarding short-term medical missions may also be due to major variations in research methodology. Varying methodologies in cost-effectiveness studies in global surgery has made it increasingly difficult to interpret and utilize results [19]. This is echoed by Nolte and authors which found that several cost-effectiveness studies for short-term surgical trips fail to adhere entirely to WHO-CHOICE recommendations, making it difficult to compare their results to other interventions [22]. It is recommended that cost-effectiveness analyses of surgical mission trips be standardized to have results more comparable to other surgical alternatives [21].

These recommendations are critical for organizations like Esperança where conducting a cost-effective analysis while entirely adhering to the WHO-CHOICE recommendations was not possible. This is because Esperanca’s current data and previous experience with evaluating the cost-effectiveness of their surgical program was limited. However, this may be a larger issue experienced by non-governmental organizations (NGO) everywhere that provide charitable medical care. There remains a lack of evidence regarding the cost-effectiveness of charitable NGO surgical platforms compared to other surgical delivery platforms in LMICs [2]. More research is necessary to evaluate the cost-effectiveness of charitable surgery delivery [2].

This study has multiple strengths to recognize. First, this study utilized patient level surgical data from multiple mission trips in recent years. The use of patient level data collected from a non-governmental organization allowed for a true evaluation of short-term global surgery interventions. Second, this study used a micro-costing approach which provided a specific and realistic breakdown of the types and value of costs associated with this intervention. Lastly, the use of a higher discount rate, which may be more applicable for the country where this intervention occurred, increases the contextual relevancy of these results.

This report should also be considered in light of multiple limitations. First, the surgical program data provided by Esperança is limited. The number of trips included in this analysis was relatively small, with only 16 surgical mission trips being included. Second, the implementation period for this intervention was relatively short. Esperança only began recording detailed patient and cost data on their surgical missions in 2016, therefore the analysis could only be conducted over these five years. Furthermore, long-term outomes for patients treated within the implementation period were not followed which may overestimate the benefits to patients. With out long-term patient data, potential future procedure complications or necessary additional procedures are not considered. Third, the analytic perspective of this analysis is from the provider perspective. The lack of data limited the ability to determine the costs to the remainder of society, the patients and their caregivers [11,12]. The provider perspective does not give a complete representation of all the costs endured to members of society such as the health care system, providers, patients, and patient caregivers. Provider costs included in this study also do not include opportunity costs which may impact the findings of the study. Currently, Esperança’s current data collection and evaluation efforts calculate providers donated services but not opportunity costs. Provider opportunity costs should be considered in similar analyses, highlighting another area of improvement for data collection and evaluation among NGOs conducting short-term medical missions. Fourth, patient data from surgical mission reports were initially in Spanish and only listed the surgical procedure that patients received. Surgical procedures were translated to English using Google Translate and may have been inaccurate. Additionally, patient data did not include the patient’s health condition diagnosis but only the surgical procedure conducted. Surgical procedures were researched to choose the most likely associated health condition for DALY calculations. The estimation of the health condition may be inaccurate, subsequently making the selected health condition and then calculated DALYs incorrect as well. Lastly, the result of this analysis was in the form of an average CER. Ideally, an incremental cost-effectiveness ratio (ICER) would have been calculated to compare Esperança’s surgical program to a counterfactual or the status quo. To calculate an ICER in this study, significant data would be necessary to determine the costs and impacts of the counterfactual. The counterfactual here would measure the costs and impacts of what intervention would otherwise take place if the surgical program did not. Because patients treated by Esperança come from various locations and required varying treatments, determining costs and impacts for how patients would have been treated or if they would not have received treatement without Esperança’s volunteer program was beyond the scope and capabilities of this research team. Differences in an average CER and an ICER can be significant [10], therefore it is possible that using an ICER as the measure of effectiveness could change the results of this analysis. This major limitation highlights the need for improved data collection efforts and capacity for evaluation among NGOs providing charitable surgical platforms as previously mentioned.

## CONCLUSIONS

Additional research methodologies are necessary for future evaluations of the charitable NGO sector and future research in this area should be structured to address fragmentation in practices and effectiveness in non-governmental organizations providing surgical interventions [2]. The results of this analysis align with these recommendations and may serve as a case study for economic evaluations of NGO charitable medical services. Future studies should examine the capacity for NGOs to conduct rigorous economic evaluations and establish best practices for how NGOs should collect data and what methodologies should be used to rigorously evaluate their data. This is crucial to improve economic evaluations of NGO charitable medical services and inform researchers and practitioners working to scale up medical services in LMICs.

## Additional material

Online Supplementary Document
