# Supplementary material for: Short-term general, gynecologic, orthopedic, and pediatric surgical mission trips in Nicaragua: A cost-effectiveness analysis
Source: J Glob Health. 2021 May 22;11:04024. doi: 10.7189/jogh.11.04024 (PMC8294827; doi:10.7189/jogh.11.04024)
Supplement: Online Supplementary Document [file jogh-11-04024-s001.pdf]

Appendix S1. Disability Weights for Calculating Disability-Adjusted Life Years (DALYs)

Table S1: Disability Weights Used to Calculate Disability-Adjusted Life Years (DALYs) for General Surgery Procedures

Procedure	Sequela	Health State	DW (95% CI)
Abscess drainage	Open wound (short term, with or without treatment)	Open wound (short term, with or without treatment)	0.006 (0.002-0.012)[1]
Anal Polypectomy	Symptomatic other skin and subcutaneous diseases	Disfigurement, level 1	0.011 (0.005-0.021)[2-3]
Appendectomy (open or laparoscopic)	Appendicitis	Abdominopelvic problem, severe	0.324 (0.22-0.442)[2]
Breast biopsy	Symptomatic other skin and subcutaneous diseases	Disfigurement, level 1	0.011 (0.005-0.021) [2-3]
Cholecystectomy (open or laparoscopic)	Moderate symptomatic episodes gallbladder and biliary diseases	Abdominopelvic problem, moderate	0.114 (0.078-0.159) [2]
Circumcision	N/A	Abdominopelvic problem, moderate	0.123 (0.083-0.176) [1]
Cryptorchidism	Undescended testes	N/A	0.317 (0.020-0.420)[4]
Cyst excision	Symptomatic other skin and subcutaneous diseases	Disfigurement, level 1	0.011 (0.005-0.021) [2-3]
Fibrolipoma excision	Symptomatic other skin and subcutaneous diseases	Disfigurement, level 1	0.011 (0.005-0.021) [2-3]
Foreign body extraction	Symptomatic other skin and subcutaneous diseases	Disfigurement, level 1	0.011 (0.005-0.021) [2-3]
Gynecomastia excision	Symptomatic other skin and subcutaneous diseases	Disfigurement, level 1	0.011 (0.005-0.021) [2-3]
Hemorrhoidectomy	N/A	N/A	0.012 N/A[5]
Hernioplasty (inguinal, umbilical, and epigastric)	Moderate symptomatic inguinal, femoral and abdominal hernia	Abdominopelvic problem, moderate	0.114 (0.078-0.159)[2]
Herniorrhaphy (inguinal, umbilical, and epigastric)	Moderate symptomatic inguinal, femoral and abdominal hernia	Abdominopelvic problem, moderate	0.114 (0.078-0.159)[2]
Hydrocelectomy	Hydrocele due to lymphatic filariasis	Epididymo-orchitis	0.128 (0.086-0.18)[2]
Incisional biopsy	Symptomatic other skin and subcutaneous diseases	Disfigurement, level 1	0.011 (0.005-0.021) [2-3]
Lipoma excision	Symptomatic other skin and subcutaneous diseases	Disfigurement, level 1	0.011 (0.005-0.021) [2-3]
Mandibular chondroma excision	Symptomatic other skin and subcutaneous diseases	Disfigurement, level 1	0.011 (0.005-0.021) [2-3]
Mass excision	Symptomatic other skin and subcutaneous diseases	Disfigurement, level 1	0.011 (0.005-0.021) [2-3]
Mastectomy	Mastectomy due to breast cancer	Mastectomy	0.036 (0.02-0.057)[6]
Sphincterotomy	Anal fissure	N/A	0.005 N/A [1]
Surgical wash	Open wound (short term, with or without treatment)	Open wound (short term, with or without treatment)	0.006[1]
Tyrosytic cyst excision	Symptomatic other skin and subcutaneous diseases	Disfigurement, level 1	0.011 (0.005-0.021) [2-3]
Varicocele Extraction	Hydrocele due to lymphatic filariasis	Epididymo-orchitis	0.128 (0.086-0.18) [2]

Wart excision	Open wound (short term, with or without treatment)	Open wound (short term, with or without treatment)	0.006 (0.002-0.012) [1]
Intradural Extramedullary Spinal Tumors	Other benign and in situ neoplasms	N/A	N/A [2]
Lymph Node excision	Other benign and in situ neoplasms	N/A	N/A [2]
Neoplasia	Other benign and in situ neoplasms	N/A	N/A [2]
Nodule excision	Other benign and in situ neoplasms	N/A	N/A [2]
Thyroidectomy	Other benign and in situ neoplasms	N/A	N/A [2]

Table S2: Disability Weights Used to Calculate Disability-Adjusted Life Years (DALYs) for Pediatric Surgery Procedures

Procedure	Sequela	Health State	DW (95% CI)
Aneurysm excision	Mild other cardiovascular diseases	Heart failure, mild	0.041 (0.026-0.062) [2]
Cholecystectomy (open or laparoscopic)	Moderate symptomatic episodes gallbladder and biliary diseases	Abdominopelvic problem, moderate	0.114 (0.078-0.159)[2]
Circumcision	N/A	Abdominopelvic problem, moderate	0.123 (0.083-0.176) [1]
Cyst excision	Symptomatic other skin and subcutaneous diseases	Disfigurement, level 1	0.011 (0.005-0.021) [2-3]
Epididymal cyst drainage	Hydrocele due to lymphatic filariasis	Epididymo-orchitis	0.128 (0.086-0.18) [2]
Fistulectomy	Anal fissure/abscess/fistula	N/A	0.082 (0.066-0.1)[7]
Herniorrhaphy	Moderate symptomatic inguinal, femoral and abdominal hernia	Abdominopelvic problem, moderate	0.114 (0.078-0.159)[2]
Hydrocelectomy	Hydrocele due to lymphatic filariasis	Epididymo-orchitis	0.128 (0.086-0.18) [2]
Lipoma excision	Symptomatic other skin and subcutaneous diseases	Disfigurement, level 1	0.011 (0.005-0.021) [2-3]
Mass excision	Symptomatic other skin and subcutaneous diseases	Disfigurement, level 1	0.011 (0.005-0.021) [2-3]
Myxoma excision	Mild other cardiovascular diseases	Heart failure, mild	0.041 (0.026-0.062) [2]
Orchidectomy	Undescended testes	N/A	0.317 (0.020-0.420) [4]
Thyroglossal cyst excision	Symptomatic other skin and subcutaneous diseases	Disfigurement, level 1	0.011 (0.005-0.021) [2-3]
Wart excision	Open wound (short term, with or without treatment)	Open wound (short term, with or without treatment)	0.006 (0.002-0.012) [1]

Table S3: Disability Weights Used to Calculate Disability-Adjusted Life Years (DALYs) for Gynecology Surgery Procedures

Procedure	Sequela	Health State	DW (95% CI)
Hysterectomy (abdominal and vaginal)	N/A	N/A	0.225 N/A [1]
Adhesion release	Mild other gynecological	Abdominopelvic	0.011

	disorders	problem, mild	(0.005-0.021) [2]
Bartholin Cyst Marsupialization	Mild other gynecological disorders	Abdominopelvic problem, mild	0.011 (0.005-0.021) [2]
Bilateral fimbriectomy	Mild other gynecological disorders	Abdominopelvic problem, mild	0.011 (0.005-0.021) [2]
Bilateral tubal occlusion (OTB)	N/A	N/A	0.238 N/A [5]
Burch	Stress incontinence due to genital prolapse	Stress incontinence	0.02 (0.011-0.035) [2]
Caesarean	Obstructed labor, acute event	Abdominopelvic problem, severe	0.324 (0.220-0.442)[2-3]
Cervical conization	Mild other gynecological disorders	Abdominopelvic problem, mild	0.011 (0.005-0.021) [2]
Colpoplasty/Colpocleisis	Stress incontinence due to genital prolapse	Stress incontinence	0.02 (0.011-0.035) [2]
Colporrhaphy (anterior and posterior)	Stress incontinence due to genital prolapse	Stress incontinence	0.02 (0.011-0.035) [2]
Cystoscopy	Mild other gynecological disorders	Abdominopelvic problem, mild	0.011 (0.005-0.021) [2]
Exploratory ovarian laparotomy	Mild other gynecological disorders	Abdominopelvic problem, mild	0.011 (0.005-0.021) [2]
Fistula repair	Obstetric fistula	N/A	0.346 (0.232–0.479) [7]
IUD insertion	Mild other gynecological disorders	Abdominopelvic problem, mild	0.011 (0.005-0.021) [2]
Jj catheter removal	Mild other gynecological disorders	Abdominopelvic problem, mild	0.011 (0.005-0.021) [2]
Oferectomy	Mild other gynecological disorders	Abdominopelvic problem, mild	0.011 (0.005-0.021) [2]
Overy cauterization	Mild other gynecological disorders	Abdominopelvic problem, mild	0.011 (0.005-0.021) [2]
Perineoplasty	Mild other gynecological disorders	Abdominopelvic problem, mild	0.011 (0.005-0.021) [2]
Perineorraphy	Mild other gynecological disorders	Abdominopelvic problem, mild	0.011 (0.005-0.021) [2]
Salpingooferectomy/Tubal removal	Ectopic Pregnancy	Abdominopelvic problem, moderate	0.114 (0.078-0.159)[2,8]
Suspension of uterus/cupula	Stress incontinence due to genital prolapse	Stress incontinence	0.02 (0.011-0.035) [2]
Tension-free vaginal tape (TVT)	Stress incontinence due to genital prolapse	Stress incontinence	0.02 (0.011-0.035) [2]
Transobturator tape (TOT)	Stress incontinence due to genital prolapse	Stress incontinence	0.02 (0.011-0.035) [2]
Ovary teratoma extraction	Benign and in situ cervical and uterine neoplasms	N/A	N/A [2]

Table S4: Disability Weights Used to Calculate Disability-Adjusted Life Years (DALYs) for Orthopedic Surgery Procedures

Procedure	Sequela	Health State	DW (95% CI)
Achilles tendon repair/elongation	Other injuries of muscle and tendon (includes sprains, strains and dislocations other than shoulder, knee, hip)	Other injuries of muscle and tendon (includes sprains, strains and dislocations other than shoulder, knee, hip)	0.008 (0.003-0.015) [6]

Adductor tenotomy	Other injuries of muscle and tendon (includes sprains, strains and dislocations other than shoulder, knee, hip)	Other injuries of muscle and tendon (includes sprains, strains and dislocations other than shoulder, knee, hip)	0.008 (0.003-0.015) [6]
Anterior cruciate ligament reconstruction	Moderate osteoarthritis of the knee	Musculoskeletal problems, lower limbs, moderate	0.079 (0.054-0.11)[2,9]
Arthrodesis (ankle)	Other musculoskeletal disorders severity level 1	Musculoskeletal problems, lower limbs, mild	0.023 (0.013-0.037) [6]
Arthroscopy	Other injuries of muscle and tendon (includes sprains, strains and dislocations other than shoulder, knee, hip)	Other injuries of muscle and tendon (includes sprains, strains and dislocations other than shoulder, knee, hip)	0.008 (0.003-0.015) [6]
Bankart procedure	Dislocation of shoulder (long term, with or without treatment)	Dislocation of shoulder (long term, with or without treatment)	0.062 (0.041-0.088) [6]
Bunionectomy	Other musculoskeletal disorders severity level 1	Musculoskeletal problems, lower limbs, mild	0.023 (0.013-0.037) [6]
Closed reduction (arm)	Fracture of radius or ulna (short term, with or without treatment)	Fracture of radius or ulna (short term, with or without treatment)	0.028 (0.016-0.046) [6]
Elbow tripceptoplasty	Fracture of radius or ulna (short term, with or without treatment)	Fracture of radius or ulna (short term, with or without treatment)	0.028 (0.016-0.046) [6]
Endomedullary cleavage (Tibia)	Fracture of patella, tibia or fibula or ankle (short term, with or without treatment)	Fracture of patella, tibia or fibula or ankle (short term, with or without treatment)	0.05 (0.032-0.075) [6]
Excision (clavicle)	Other musculoskeletal disorders severity level 2	Musculoskeletal problems, upper limbs, mild	0.028 (0.017-0.045) [2]
Extraction of osteonosis material (radius)	Other musculoskeletal disorders severity level 2	Musculoskeletal problems, upper limbs, mild	0.028 (0.017-0.045) [2]
Extraction of osteonosis material (tibia)	Other musculoskeletal disorders severity level 1	Musculoskeletal problems, lower limbs, mild	0.023 (0.013-0.037) [6]
Finger osteoarthritis repair	Other musculoskeletal disorders severity level 2	Musculoskeletal problems, upper limbs, mild	0.028 (0.017-0.045) [2]
Finger plasty	Other musculoskeletal disorders severity level 2	Musculoskeletal problems, upper limbs, mild	0.028 (0.017-0.045) [2,10]
Flexor tenorrhaphy (hand)	Other musculoskeletal disorders severity level 2	Musculoskeletal problems, upper limbs, mild	0.028 (0.017-0.045) [2,10]
Hallux Valgus correction	Other musculoskeletal disorders severity level 1	Musculoskeletal problems, lower limbs, mild	0.023 (0.013-0.037) [6]
Hand/finger arthrodesis	Other musculoskeletal disorders severity level 2	Musculoskeletal problems, upper limbs,	0.028 (0.017-0.045) [2]

		mild	
Hip hemi-plasty	Fracture of neck of femur (short term, with or without treatment)	Fracture of neck of femur (short term, with or without treatment)	0.258 (0.172-0.356) [6]
Infrachondilar amputation (lower limb)	Amputation of one lower limb (long term, without treatment)	Amputation of one lower limb (long term, without treatment)	0.173 (0.118-0.240) [6]
Mass excision (finger)	N/A	Disfigurement: level 1 with itch or pain	0.029 0.016-0.048[10]
Meniscectomy	Moderate osteoarthritis of the knee	Musculoskeletal problems, lower limbs, moderate	0.079 (0.054-0.11)[2,9]
Meniscus laxity	Moderate osteoarthritis of the knee	Musculoskeletal problems, lower limbs, moderate	0.079 (0.054-0.11)[6,9]
Nerve rearrangement	Injured nerves (short term)	Injured nerves (short term)	0.1 (0.067-0.14) [6]
Nerve release	Injured nerves (short term)	Injured nerves (short term)	0.1 (0.067-0.14) [6]
Open reduction and internal fixation (Femur)	Fracture, other than femoral neck (short term, with or without treatment)	Fracture, other than femoral neck (short term, with or without treatment)	0.111 (0.074-0.156) [6]
Open reduction and internal fixation (Hand)	Fracture of hand (short term, with or without treatment)	Fracture of hand (short term, with or without treatment)	0.01 (0.005-0.019) [6]
Open reduction and internal fixation (Hip)	Fracture of neck of femur (short term, with or without treatment)	Fracture of neck of femur (short term, with or without treatment)	0.258 (0.172-0.356) [6]
Open reduction and internal fixation (Humerous)	Fracture of clavicle, scapula or humerus (short or long term, with or without treatment)	Fracture of clavicle, scapula or humerus (short or long term, with or without treatment)	0.035 (0.021-0.053) [6]
Open reduction and internal fixation (Knee)	Fracture of patella, tibia or fibula or ankle (short term, with or without treatment)	Fracture of patella, tibia or fibula or ankle (short term, with or without treatment)	0.05 (0.032-0.075) [6]
Open reduction and internal fixation (Patela)	Fracture of patella, tibia or fibula or ankle (short term, with or without treatment)	Fracture of patella, tibia or fibula or ankle (short term, with or without treatment)	0.05 (0.032-0.075) [6]
Open reduction and internal fixation (Radius)	Fracture of radius or ulna (short term, with or without treatment)	Fracture of radius or ulna (short term, with or without treatment)	0.028 (0.016-0.046) [6]
Open reduction and internal fixation (Scapula)	Fracture of clavicle, scapula or humerus (short or long term, with or without treatment)	Fracture of clavicle, scapula or humerus (short or long term, with or without treatment)	0.035 (0.021-0.053) [6]
Open reduction and internal fixation (Tibia)	Fracture of patella, tibia or fibula or ankle (short term, with or without treatment)	Fracture of patella, tibia or fibula or ankle (short term, with or without treatment)	0.05 (0.032-0.075) [6]
Patella dislocation repair	Dislocation of knee (long term, with or without treatment)	Dislocation of knee (long term, with or without treatment)	0.113 (0.075-0.160) [6]

Patella reconstruction	Fracture of patella, tibia or fibula or ankle (short term, with or without treatment)	Fracture of patella, tibia or fibula or ankle (short term, with or without treatment)	0.05 (0.032-0.075) [6]
Polydactyly Excision	Disfigurement level 1 due to polydactyly and syndactyly	Disfigurement, level 1	0.011 (0.005-0.021) [2]
Pseudarthrosis correction	Fracture of vertebral column (short or long term, with or without treatment)	Fracture of vertebral column (short or long term, with or without treatment)	0.111 (0.075-0.156) [6]
Radius head recession	Fracture of radius or ulna (short term, with or without treatment)	Fracture of radius or ulna (short term, with or without treatment)	0.028 (0.016-0.046) [6]
Release of tendonitis (carpal)	Injured nerves (short term)	Injured nerves (short term)	0.1 (0.067-0.14) [6,9]
Repair of radial and humeral ligament	Other injuries of muscle and tendon (includes sprains, strains and dislocations other than shoulder, knee, hip)	Other injuries of muscle and tendon (includes sprains, strains and dislocations other than shoulder, knee, hip)	0.008 (0.003-0.015) [6]
Rotator cuff reconstruction	Dislocation of shoulder (long term, with or without treatment)	Dislocation of shoulder (long term, with or without treatment)	0.062 (0.041-0.088) [6]
Sub-scapular translocation	Dislocation of shoulder (long term, with or without treatment)	Dislocation of shoulder (long term, with or without treatment)	0.062 (0.041-0.088) [6]
Supracondylar amputation of left limb	Amputation of one lower limb (long term, without treatment)	Amputation of one lower limb (long term, without treatment)	0.173 (0.118-0.240) [6]
Surgical/arthroscopic wound debridement	Other injuries of muscle and tendon (includes sprains, strains and dislocations other than shoulder, knee, hip)	Other injuries of muscle and tendon (includes sprains, strains and dislocations other than shoulder, knee, hip)	0.008 (0.003-0.015) [6]
Tendon contracture plasty	Other injuries of muscle and tendon (includes sprains, strains and dislocations other than shoulder, knee, hip)	Other injuries of muscle and tendon (includes sprains, strains and dislocations other than shoulder, knee, hip)	0.008 (0.003-0.015) [6]
Tendon contracture plasty	Other injuries of muscle and tendon (includes sprains, strains and dislocations other than shoulder, knee, hip)	Other injuries of muscle and tendon (includes sprains, strains and dislocations other than shoulder, knee, hip)	0.008 (0.003-0.015) [6]
Tibia scar tissue release	Other musculoskeletal disorders severity level 1	Musculoskeletal problems, lower limbs, mild	0.023 (0.013-0.037)[2,10]
Unstable glenohumeral dislocation	Dislocation of shoulder (long term, with or without treatment)	Dislocation of shoulder (long term, with or without treatment)	0.062 (0.041-0.088) [6]
Intradural extramedullary (IDEM)	Other benign and in situ neoplasms	N/A	N/A [2]

References

1. Chatterjee S, Laxminarayan R, Gosselin RA. Cost Per DALY Averted in a Surgical Unit of a Private Hospital in India. *World J Surg*. 2016;40(5):1034-1040.
2. Global Burden of Disease Collaborative Network. Global Burden of Disease Study 2017 (GBD 2017) Disability Weights. Seattle, United States: Institute for Health Metrics and Evaluation (IHME), 2018.
3. Aung EE, Liabsuetrakul T, Panichkriangkrai W, Makka N, Bundhamchareon K. Years of healthy life lost due to adverse pregnancy and childbirth outcomes among adolescent mothers in Thailand. *AIMS Public Health*. 2018;5(4):463-476.
4. Poenaru D, Lin D, Corlew S. Economic Valuation of the Global Burden of Cleft Disease Averted by a Large Cleft Charity. World J Surg. 2016 May;40(5):1053-9.
5. Egle JP, McKendrick A, Mittal VK, Sosa F. Short-term surgical mission to the Dominican Republic: a cost-benefit analysis. *Int J Surg*. 2014;12(10):1045-1049.
6. Global Burden of Disease Collaborative Network. Global Burden of Disease Study 2015 (GBD 2015) Disability Weights. Seattle, United States: Institute for Health Metrics and Evaluation (IHME), 2016.
7. Higashi H, Barendregt JJ, Kassebaum NJ, Weiser TG, Bickler SW, Vos T. Surgically avertable burden of obstetric conditions in low- and middle-income regions: a modelled analysis. *BJOG*. 2015;122(2):228-236.
8. Grimes CE, Henry JA, Maraka J, Mkandawire NC, Cotton M. Cost-effectiveness of surgery in low- and middle-income countries: a systematic review. *World J Surg*. 2014;38(1):252-263.
9. Chen AT, Pedtke A, Kobs JK, Edwards GS Jr, Coughlin RR, Gosselin RA. Volunteer orthopedic surgical trips in Nicaragua: a cost-effectiveness evaluation. *World J Surg*. 2012;36(12):2802-2808.
10. Tadisina KK, Chopra K, Tangredi J, Thomson JG, Singh DP. Helping hands: a cost-effectiveness study of a humanitarian hand surgery mission. *Plast Surg Int*. 2014;2014:921625.

Appendix S2. Example of Calculations for DALYs

For this example, the patient received care during a general surgery mission trip. The patient was a 44-year-old female who received a laparoscopic cholecystectomy. Below is the equation.

The associated health condition for a laparoscopic cholecystectomy is moderate symptomatic episodes of gallbladder and biliary diseases, a moderate abdominopelvic problem in which the patient likely has pain in the belly, feels nauseous, and has difficulties with daily activities. The associated disability weight (DW) is 0.114. This information and DW were obtained from the 2017 Global Burden of Disease Study.

The age-specific life expectancy for a 44-year-old female in Nicaragua is 41.4. This value was obtained from the WHO Life Tables for Nicaragua.[1]

$$\text{DALYs Averted} = \frac{\mathrm{I} \cdot \mathrm{DW} \cdot (1 - e^{-rL})}{r}$$

I = incidence
DW = disability weight
r = discount rate
L = age-specific life expectancy

For this case:

I = 1
DW = 0.114
r = 0.03
L = 41.4

$$\text{DALYs Averted} = \frac{1 \cdot 0.114 \cdot (1 - e^{(-0.03)(41.4)})}{0.03} = 2.7025$$

As seen in the equation above, this surgical procedure averted 2.7025 DALYs for this patient.

Reference

1. World Health Organization. Life Tables by Country Nicaragua. Available from: https://apps.who.int/gho/data/view.main.61180?lang=en. [Accessed July 31, 2020].
